# Functions of standard CPR training on performance qualities of medical volunteers for Mt. Taishan International Mounting Festival

**DOI:** 10.1186/1471-227X-13-S1-S3

**Published:** 2013-07-04

**Authors:** Meng Fanshan, Zhao Lin, Liu Wenqing, Lu Chunlei, Liu Yongqiang, Li Naiyi

**Affiliations:** 1 Emergency Department, the 88th Hospital of PLA, Taian Shandong Province, 271000,China

## Abstract

**Background:**

Cardiopulmonary resuscitation (CPR) is a sudden emergency procedure that requires a rapid and efficient response, and personnel training in lifesaving procedures. Regular practice and training are necessary to improve resuscitation skills and reduce anxiety among the staff. As one of the most important skills mastered by medical volunteers serving for Mt. Taishan International Mounting Festival, we randomly selected some of them to evaluate the quality of CPR operation and compared the result with that of the untrained doctors and nurses. In order to evaluate the functions of repeating standard CPR training on performance qualities of medical volunteers for Mt. Taishan International Mounting Festival, their performance qualities of CPR were compared with those of the untrained medical workers working in emergency departments of hospitals in Taian.

**Methods:**

The CPR performance qualities of 52 medical volunteers (Standard Training Group), who had continually taken part in standard CPR technical training for six months, were tested at random and were compared with those of 68 medical workers (Compared Group) working in emergency departments of hospitals in Taian who hadn’t attended CPR training within a year. The QCPR 3535 monitor (provided by Philips Company) was used to measure the standard degree of single simulated CPR performance, including the chest compression depth, frequency, released pressure between compressions and performance time of compression and ventilation, the results of which were recorded in the table and the number of practical compression per minute was calculated. The data were analyzed by x2 Test and t Test. The factors which would influence CPR performance, including gender, age, placement, hand skill, posture of compression and frequency of training, were classified and given parameters, and were put to Logistic repression analysis.

**Results:**

The CPR performance qualities of volunteers were much higher than those of the compared group. The overall pass rates were respectively 86.4% and 31.9%; the pass rates of medical volunteers in terms of the chest compression depth, frequency, released pressure between compressions were higher than those of the compared group, which were 89.6%, 94.2%, 95.8% vs 50.3%, 53.0%, 83.1%, P<0.01; there were few differences in overall performance time, which were (118.4±13.5s) vs (116.0±10.4s), P>0.05; the duration time of ventilation in each performance section was much shorter than that in the compared group, which were (6.38±1.2) vs (7.47±1.7), P<0.01; there were few differences in the number of practical compression per minute, which were (78.2±3.5) vs (78.8±12.2), P>0.05); the time proportion of compression and ventilation was 2.6:1 vs 2.1:1. The Logistic repression analysis showed that CPR performance qualities were clearly related to hand skill, posture of compression and repeating standard training, which were respectively OR 13.12 and 95%CI (2.35~73.2); OR 30.89, 95%CI (3.62~263.5); OR 4.07,95%CI (1.16~14.2).

**Conclusion:**

The CPR performance qualities of volunteers who had had repeating standard training were much higher than those of untrained medical workers, which proved that standard training helped improve CPR performance qualities.

## 

The medical volunteers serving for Mt. Taishan International Mounting Festival needed to take part in various first-aid technical training, among which CPR was the most important. In order to know the practical CPR performance qualities and the functions of training, CPR performance of medical volunteers was tested at random, and the results were compared with those of ordinary medical workers working in the emergency department of 6 hospitals in Taian to evaluate the functions of standard CPR training and to learn the qualities of CPR performance of medical volunteers in potential important events.

## Materials and methods

### Objects of investigation

In May, 2012 52 healthy medical volunteers coming from hospitals in Taian were selected at random according to their card numbers. The criterion of selection was that the objects had had repeating standard CPR training of basic theories and techniques in the past 6 months. Among them there were 36 males and 16 females. The proportion of males and females was 2.15:1; the proportion of doctors and nurses was 2.1:1; the average age was 34±4.5 years, and the average working time was 7±3.8 years. The compared group was composed of 68 medical workers selected at random from those who had been working in the emergency department of 3 hospitals in Taian according to their card numbers. The criterion of selection was that the objects hadn’t had any standard CPR training within a year. Among them there were 45 males and 23 females. The proportion of males and females was 1.96:1; the proportion of doctors and nurses was 2:1; the average age was 34±5.5 years, and the average working time was 8±4.6 years. There were few statistic differences between the two groups.

### Index of performance qualities

A testee’s simulated performance of mouth-to-mouth ventilation and out-of-chest heart compression was measured. The proportion of compression and ventilation was 30:2, according to 2010 AHA CPR Guideline[1]. The performance time was recorded at the beginning of heart compression, and ended until 5 sets of 30:2 compression and ventilation were finished. And the respective time of each set was recorded and calculated by second with the help of the equipment. The main indexes of qualities were performance process, hand skill, posture, placement, depth, frequency and released pressure between compressions.

## Methods

The QCPR3535 monitor was used to measure and record each performer’s heart compression depth, frequency, released pressure between compressions and the time of compression and ventilation, which was supervised by pressure distance sensor placed on chest under the hands, and the hand skill, position and posture were recorded. The measured data were evaluated by 2 persons, one of whom recorded them into the table and fed them into the computer, and the other checked to make sure the data were accurate and reliable.

### Testing instruments

The QCPR3535 monitor with a pressure distance sensor which could be placed on the sternum was provided by Philips Company. The sensor could gather the data, and deliver them to HeartStart MRx to be explained. The wave table could be used to show compression frequency and depth. The height of waves showed the compression depth, and the time interval between waves showed the frequency and the data above waves were the calculated number of compression per minute. The equipment could make real-time analysis of the compression, and compare the practical performance with that of the AHA Guideline 2010. If the compression depth or frequency exceeded the target, MRX would show signals on the screen and provide feedback sound. The compression depth was tested by compression depth waves on the monitor, and if the depth exceeded the line, it was proper. If the depth couldn’t reach the line, it was too low; the proper compression frequency was no less than 100 times per minute. The released pressure between compressions could be shown with special signals on the screen, and it could be shown by the “*”s on the monitor screen. If there was “*”on the screen, that meant no pressure was released; if there was no “*”on the screen, that meant pressure was released. The same Annier CPR simulated dummies were used in the test.

### Statistical processing

The SPSS15.0 software system was used in data processing. The measured materials were expressed with x- ± s, and analyzed by t Test; the calculated materials were tested by χ2, and the influence factors were analyzed by multi-factor Logistic repression method, and P<0.05 referred to the distinct difference in statistics.

## Results

According to the testing indexes of compression depth, frequency and released pressure between compressions, 86.4% of the medical volunteers met the standard requirements of performance, and only 31.9% of the medical workers in the compared group could meet the standard requirements of performance. In terms of tested unilateral indexes, the pass rate of volunteers was much higher than that of ordinary medical workers, as could be shown in Table 1.

**Table 1:** Comparison of Two Groups in Performance Qualities

Group	n	Depth		Frequency		Released Pressure	
		
		Proper	Lower	Proper	Slower	Y	N
Volunteers Control	52 68	46(88.5%) 38(55.9%)	6(11.5%) 30(44.1%)	49(94.2%) 42(61.8%)	3(5.8%) 26(38.2%)	50(95.5%) 37(54.4%)	2(4.5%) 31(45.6%)
*X^2^* *P*		23.27 <0.01		33.80 <0.01		31.40 <0.01	

There were no distinct differences between the two groups in the average number of practical compression per minute. Compared with that of ordinary medical workers, the time of ventilation used by the volunteers was shorter. There was no difference between the two groups in the total time used in 5 CPR, which were 118.4±18.5s and 116.0±5.4s. There was no distinct difference between the two groups in the number of practical compression per minute, as could be shown in Table 2.

**Table 2:** Comparison of Number of Practical Compression Per Minute & Time of Compression and Ventilation (x- ± s)

Group	n	Practical Compression per Minute (n/min)	Ventilation Time of Each Set(S)	Time Proportion of Compression and Ventilation
Volunteers Control	52 68	78.2±3.5 78.8±12.2	6.38±1.2* 7.47±1.7	2.6:1 2.1:1

Note: Compared with those ordinary medical workers/ doctors t=4.41, ^*^P<0.05

The factors which could influence CPR performance were given parameters, including gender (male=0, female=1) and age (≤30y=0, 31y~45y=1, ≥46y=2); proper hand skill, posture and position, and attending repeating standard training were given 0, and on the contrary they were given 1.The tested results of multi-factor parameters were put into Logistic Repression Model and analyzed. The results showed that such factors as the hand skill, posture of compression and repeating training had clear relationship with CPR performance qualities, as could be shown in Table 3.

**Table 3:** Logistic Repression Analysis of CPR Performance

Parameters	*B*	Standard Error	u	*P*	*OR*	95%*CI*
Constant Item Hand Skill Posture Standard Training Age Placement Gender	-1.760 2.644 4.030 1.613 0.015 -0.409 -0.112	1.645 0.867 1.104 0.642 0.049 1.104 0.505	1.070 2.956 3.235 2.208 0.305 0.476 0.205	0.2848 0.0031 0.0016 0.0207 0.6923 0.6317 0.8230	14.02 32.85 4.76 0.934 0.453 0.809	2.340~72.245 3.661~265.578 1.189~15.427 0.907~1.004 0.061~4.317 0.287~2.430

## Discussion

CPR is one of the most important emergency techniques. Proper, immediate and effective CPR is the key to resuscitation, and it should be popularized as an important item in public health care. The emergency work is very important in meetings, ceremonies, sports games and so on. Before Mt. Taishan International Mounting Festival was held, the medical volunteers were experienced repeating trainings of emergency theories and techniques for six months. The study selected 52 medical volunteers at random and tested their CPR performance, and compared them with those of ordinary medical workers who had been working in emergency department in 3 hospitals of Taian. The study showed that in terms of compression depth, frequency and released pressure between compressions, the medical volunteers performed much better than those ordinary medical workers who hadn’t had systematic training, with overall pass rate being 86.4% vs 31.9%. After analyzing the performance process, the main reasons that the target hitting rate of those untrained medical workers was low were the compressions were too slow or too low, and that no pressure was released between compressions was also an important reason. The reasons for low compression frequency were the performers tried to perform well, but they were too nervous. The compression frequency of the compared group was too slow, which was 53.0%, but the average practical compression number per minute was no less than that of the volunteers. That was mainly because some of them performed so fast that they could save the time to increase the practical compression time. The compression time of the volunteers was much higher than that of the compared group, which could reduce the non-recycling time and could be more beneficial to successful resuscitation. The volunteers’ skillful performance, proper compression frequency and depth were closely related to continual standard training.

It was obvious that the medical volunteers’ group training and persistence in regular intensified training and testing could keep their CPR performance qualities at a high level and could provide high-quality service for the Mt. Taishan International Mounting Festival. The Logistic Repression analysis showed that CPR performance qualities were clearly related to such factors as hand skill, posture of compression and repeating standard training, etc., but hand skill and posture of compression were greatly influenced by repeating training. Therefore, repeating training was the most important factor in influencing CPR qualities. In order to improve CPR performance qualities, the medical workers should be trained and tested regularly and repeatedly. A study showed that the immediate information feedback after training and test after 6 weeks would greatly improve the CPR performance level, otherwise, the performance level would decrease with the time[2,3]. The CPR teaching qualities, CPR Guideline and the process of life chain were related to the survival rate of those patients who suffered from cardiac arrest[4,5]. The recent studies discovered that many medical workers couldn’t perform standard CPR, one reasons for which might be insufficient education and training [6-8]. Some other studies discovered that although a lot of work had been done, few trainees could perform CPR. Even though they performed, the qualities were not very high[9]. There was a gap between people who were most likely to be witnesses of cardiac arrest and those who had been trained [6]. Considering the influence of psychological factors, psychological training was necessary[10]. The CPR performers should be in good physical and psychological state.

The studies showed that long-distance net teaching couldn’t replace traditional CPR education, so it was necessary to add simulated training to traditional CPR training[10,11]. Because the mastery degree of CPR techniques would decrease with time, a plan of continual training system should be made. According to the studies, 17 months after the first training most of the volunteers had mastered the core techniques of CPR and AED. The researches suggested that these tests should be further studied in practice [12,13]. CPR of cardiac arrest in special situations was also suggested to be trained[14-16].The fact that the medical volunteers’ group training and persistence in regular intensified training and testing could improve their CPR performance qualities proved that it was necessary for medical workers to strengthen repeating, standard and effective training of CPR techniques, and it was the Mt. Taishan International Mounting Festival that brought the opportunity of improving CPR level to our country.

## Declarations

This article has been published as part of *BMC Emergency Medicine* Volume 13 Supplement 1, 2013: Proceedings of the 2012 Emergency Medicine Annual Congress. The full contents of the supplement are available online at http://www.biomedcentral.com/bmcemergmed/supplements/13/S1.

The publication costs for this article was funded by the 88th Hospital of PLA, Tai’an Shandong Province, 271000,China.
